# Suppression of neuronal cholesterol biosynthesis impairs brain functions through insulin-like growth factor I-Akt signaling

**DOI:** 10.7150/ijbs.63512

**Published:** 2021-08-27

**Authors:** Ting Liu, Yang Li, Baoyu Yang, Haozhen Wang, Chen Lu, Alan K Chang, Xiuting Huang, Xiujin Zhang, Ziyin Lu, Xiuli Lu, Bing Gao

**Affiliations:** 1 The School of Life Science, Liaoning University, Chongshanzhong-lu No.66, Huanggu-qu, Shenyang 110036, China; 2 China Medical University-The Queen's University Belfast Joint College, China Medical University, Shenyang, 110122, China; 3 School of Basic Medical Sciences, Shenyang Medical College, Shenyang, 110034, China

**Keywords:** Cholesterol suppression, DHCR24, IGF-1 signaling, Central neuronal system

## Abstract

Some relationship between abnormal cholesterol content and impairment of insulin/insulin-like growth factor I (IGF-1) signaling has been reported in the pathogenesis of Alzheimer's disease (AD). However, the underlying mechanism of this correlation remains unclear. It is known that 3-β hydroxycholesterol Δ 24 reductase (DHCR24) catalyzes the last step of cholesterol biosynthesis. To explore the function of cholesterol in the pathogenesis of AD, we depleted cellular cholesterol by targeting DHCR24 with siRNA (siDHCR24) or U18666A, an inhibitor of DHCR24, and studied the effect of the loss of cholesterol on the IGF-1-Akt signaling pathway in vitro and in vivo. Treatment with U18666A reduced the cellular cholesterol level and blocked the anti-apoptotic function of IGF-1 by impairing the formation of caveolae and the localization of IGF-1 receptor in caveolae of the PC12 cells. Downregulation of the DHCR24 expression induced by siRNA against DHCR24 also yielded similar results. Furthermore, the phosphorylation levels of IGF-1 receptor, insulin receptor substrate (IRS), Akt, and Bad in response to IGF-1 were all found to decrease in the U18666A-treated cells. Rats treated with U18666A via intracerebral injection also exhibited a significant decrease in the cholesterol level and impaired activities of IGF-1-related signaling proteins in the hippocampus region. A significant accumulation of amyloid β and a decrease in the expression of neuron-specific enolase (NSE) was also observed in rats with U18666A. Finally, the Morris water maze experiment revealed that U18666A-treated rats showed a significant cognitive impairment. Our findings provide new evidence strongly supporting that a reduction in cholesterol level can result in neural apoptosis via the impairment of the IGF-1-Akt survival signaling in the brain.

## Introduction

Cholesterol is one of the most important biomolecules in cell physiology due to its involvement in several significant biological processes. For example, cholesterol forms caveolae or lipid rafts that facilitate the localization of proteins, such as enzymes and growth factor receptors, in the central nervous system (CNS). Cholesterol also plays an important function in synapse plasticity 1. The CNS is different from the other peripheral organs in terms of cholesterol metabolism and requirements. The CNS cholesterol is not affected by the blood cholesterol level, but it is derived via *de novo* cholesterol biosynthesis, mainly in the glial cells. Increasing evidences suggest the relevance of abnormal CNS cholesterol metabolism to the Alzheimer's disease (AD). For instance, cerebral cholesterol is the key factor affecting the AD phenotype in some AD patients 2. Numerous studies of the brain samples from AD-affected individual have demonstrated reduced cholesterol levels in structures such as the hippocampus 3-5. Furthermore, the mRNA level of 3β-hydroxycholesterol 24 reductase (DHCR24), the enzyme that catalyzes the last step of cholesterol biosynthesis, is decreased in the affected brain areas of AD patients 6. These data strongly suggest the relationship among cholesterol, DHCR24, and AD.

Recent studies have suggested that insulin/insulin-like growth factor (IGF) signaling pathway is also impaired in the brain of AD patients. For example, it is reported that the expression levels of insulin receptor (IR) and insulin-signaling proteins were lower throughout the AD patients' brain 7. Long-term insulin therapy is reportedly effective for patients with amnestic mild cognitive impairment and for patients with AD 8. This evidence strongly demonstrated the involvement of insulin/IGF-1 signaling in the pathological mechanisms of AD. However, very little is known about the mechanism by which the insulin/IGF-1 signaling is impaired in AD patients.

We previously demonstrated that DHCR24 exerts anti-apoptotic function through the caveolae structure, thereby facilitating the insulin-Akt cascade associated with cell survival in embryonic fibroblasts 9. The cholesterol-rich caveolae may also be required for the neuron-protective function of IGF-1 in neurons, as suggested by experiments utilizing the cholesterol-chelating reagent cyclodextrin (CD) 10. However, given that CD is not a cholesterol-specific chelating reagent, it remains unclear whether the impaired IGF-1 signaling can be attributed to the loss of cholesterol in neurons. Based on these findings, we speculated that cholesterol loss induced by the downregulation of DHCR24 may contribute to the impairment of IGF-1 survival signaling, resulting in neuron apoptosis.

In the present study, we targeted DHCR24 with siRNA against DHCR24 as well as with its inhibitor (U18666A) and demonstrated that cholesterol loss resulting from targeting DHCR24 impaired the IGF-1-Akt signaling and induced apoptosis in the CNS. In addition, we reported, for the first time, that cholesterol loss caused by inhibiting its *de novo* synthesis through the inhibition or knockdown of DHCR24 resulted in memory impairment in the experimental animal model.

## Materials and Methods

### Cell Culture

The PC12 cells were purchased from the American Type Culture Collection, Manassas, VA and were cultured in DMEM/F12 medium supplemented with 10% fetal bovine serum. The cell images were obtained with a phase-contrast microscope (IMT-2; Olympus, Tokyo, Japan).

### Analysis of microarray data

Bioinformatics analysis tools were used for the identification of different expressions of DHCR24 from a microarray data in GEO, a public and freely available database. We selected the *GSE36980* expression profile, which was based on the Agilent GPL6244 ([HuGene-1_0-st] Affymetrix Human Gene 1.0 ST Array [transcript (gene) version]). The GSE36980 dataset included 26 samples from AD patients and 62 healthy control samples. We downloaded the Series Matrix File of GSE36980 from the GEO database. Next, we conducted the GEO2R function to analyze the profile graph of DHCR24 by entering the corresponding identifier from the ID column of the Platform record. Data from the GSE36980 validate AD patients and the controls value of the DHCR24 in the hippocampi and frontal, temporal cortices, hippocampi respectively.

### Experimental model

Adult Sprague Dawley rats (weight: 250-300 g) were randomly chosen for U18666A-treated group (n=24) and for the saline control group (n=16). These rats were anesthetized via an intraperitoneal injection of 4% chloral hydrate and subsequently administered with bilateral intracerebroventricular (ICV) injection of saline or U18666A ((3-β-3-[2-(Diethyl amino) ethoxy] androst-5-en-17-one hydrochloride; Sigma-Aldrich, St. Louis, MO; 10 μL). U18666A was delivered at 1.0-mm posterior and 1.7-mm lateral to the bregma and 3.8-mm from the skull surface of each hemisphere using a 30-gauge needle affixed to the Hamilton Microliter Syringe. Preliminary studies have demonstrated that U18666A-mediated impaired Akt signaling in all doses tested (4-200 μg/kg). In the present paper, we chose 200 μg/kg U18666A as our treatment paradigm for consistency purposes. The U18666A-treated group was then divided into 3 subgroups, and the control group was divided into 2 subgroups. Each group included 8 rats, and they were sacrificed via decapitation at 0, 7, 14, and 21 days after the injection. All animals survived until they were sacrificed at 0, 7, 14, or 21 days after the injection. After sacrification at each time-point, fresh brains were weighed and then cut in the coronal plane to obtain approximately 3-mm-thick slice that flanked the infundibulum. The rats from one of the two control groups were sacrificed at 0 day and subjected to western blotting, high-performance liquid chromatography (HPLC) and immunohistofluorescent analyzes (represented by PBS in some experiments), while the other rats from another control subgroup and the U18666A-injected group for 21 days were sacrificed following the Morris water maze test after 21 days of receiving the injection. In addition, the brains obtained from rats with U18666A-injection for 14 days were also subjected to the HPLC and immunohistofluorescent analyzes. The procedures and the use of rats in these experiments were approved by the Shenyang Medical College IACUC Committee on the Care and Use of the Experimental Animals.

### Analysis of adherent cell numbers and apoptosis assay

Detached cells were removed from the culture plate, followed by the collection of adherent cells via centrifugation. The collected cells were stained with Trypan Blue by using the Trypan Blue-Staining Cell Viability Assay Kit (Biyuntian, Shanghai, China).

Cellular apoptosis was assessed through immunocytochemistry-based caspase-3 assay and *in situ* DNA fragmentation assay using the TUNEL kit. The immunocytochemical analysis was performed for detecting the active caspase-3 level, as described previously 11. Briefly, the cells were fixed and blocked, and the cells were incubated with rabbit antibody directed against active caspase-3 (Sigma-Aldrich). Several images were captured using the same set of optical parameters.

### Determination of intracellular cholesterol and desmosterol

Lipid was extracted from the cultured cells or tissues using the method of Bligh and Dyer 12. After drying, the lipid was dissolved in ethanol and subjected to high-performance liquid chromatography (HPLC) using cholesterol and desmosterol as the respective standards. Liquid chromatograph (Model LC-10A VP and LC-10A; Shimadzu, Kyoto, Japan) was equipped with a variable-wavelength UV-visible detector (Models SPD-M10A VP and SPD-10AV; Shimadzu), which was set to monitor the absorbance at 210 nm. The samples were separated through a reversed-phase chromatographic column (TSK gelODS-80Ts QA; 150 x 4.6-mm I.D., particle size 5 μm; Tosoh, Tokyo, Japan). The column temperature was maintained at 40°C. All analyzes were performed isocratically with acetonitrile-methanol as the eluent at a flow-rate of 1.0 mL/min. All chromatograms were analyzed with the Shimadzu Model CLASS-VP (ver. 5.04) software.

### Western blotting

Whole-cell lysate was prepared for western blotting as described previously 9 using the following primary antibodies: rabbit anti-cleaved caspase-3, anti-phospho-Akt (Ser473), anti-Akt, anti-phospho-Bad (Ser136), anti-Bad, anti-GSK-3β (Ser9), anti-phospho IGF-1R (p-Y1131) and anti-IGF-1R antibodies (Cell Signaling, Beverly, MA). Rabbit anti-NSE and anti-GFAP were obtained from Protein TechTM (Chicago, IL). Rabbit anti-phospho-Tau (Ser404) was obtained from Signaling Antibody (Beverly). Rabbit anti-caveolin-1 and mouse anti-IGF-IRβ subunit antibodies were purchased from Santa Cruz (Dallas, TX). Rabbit anti-phospho-insulin receptor substrate (IRS)-1 and anti-IRS antibodies were obtained from Bio Source International (Camarillo, CA). Rabbit anti-actin antibody was purchased from Sigma-Aldrich. Immunoblots were visualized with enhanced chemiluminescence (ECL) reagents (Pierce, Rockford, IL). The images of the blotted membranes were obtained with the LAS-1000 Lumino-image Analyzer (Fuji Film, Tokyo, Japan), and densitometric analysis was performed using the software for the LAS-1000.

### Immunocytochemical and immunohistofluorescent analysis

Confocal laser-scanning microscopy was performed as previously described 13. The primary antibodies of rabbit anti-caveolin-1 polyclonal antibodies were obtained from Santa Cruz Biotechnology (Santa Cruz, CA) and mouse anti-IGF-Iβ monoclonal antibody from BD Biosciences (Bedford, MA). The primary antibodies of anti-phospho-Akt (Ser473) polyclonal antibodies were obtained from Cell Signaling (Beverly) and rabbit anti-phospho-Tau (Ser9) was obtained from Signaling Antibody (Beverly). Rabbit anti-NSE and anti-GFAP were obtained from Protein TechTM (Chicago, IL). The anti-mouse IgG antibody was conjugated to Alexa fluor-488 and anti-rabbit IgG antibody was conjugated to Alexa fluor-568 (Molecular Probes, Eugene, OR). In some experiments, PC12s were also cultured in the serum-free DMEM containing 40 μg/ml cholesterol, which was prepared by diluting the stock cholesterol solution (20 mg/ml in ethanol). In some other experiments, the cells were incubated with antibodies against active caspase-3, GFAP, phospho-Akt, or phospho-Tau, followed by a secondary antibody. Images were obtained using a confocal laser microscope (LSM510; Carl Zeiss, Jena, Germany). For quantification of the fluorescence intensity, non-saturated images were obtained with a full-open pinhole. Three independent experiments were conducted, and 50 cells were randomly selected from a single group using the same set of optical parameters, and their fluorescence intensity was measured and calculated to obtain an average value per cell. The Zeiss LSM 510 Image Examiner software was used to analyze all the images.

### Morris water maze test

Behavioral examinations were performed in a Morris water maze as described in a previous study on the 21st day after the animals were treated with U188866 14. Briefly, a circular black pool (1.5-m diameter; 50-cm depth) was filled with water up to a depth of 30 cm. The water temperature was set to approximately 23°C. A clear circular platform (12-cm diameter) was submerged 2-cm underwater in the northeast quadrant of the pool, and the water was made opaque with milk. Each rat underwent 4 trials/day for 5 consecutive days. During the place navigation trial, the rats were placed in a random position in the pool, each facing the wall from a pre-set starting point. The animals were allowed to swim for a maximum of 90 s or until they located the platform. On day 5 of the experiment, the spatial probe trial was conducted, in which the platform was removed from the pool and the rats were allowed to swim for 90 s. The total swimming time (escape latency in seconds); the number of times the animal crossed the previous location of the platform (platform-crossing); the time that the animal spent in the quadrant of the platform, and the average swimming velocity were recorded using a video tracking system (SLY-WMS Morris Water Maze System; Beijing Sunny Instruments Co., Ltd., Beijing, China).

### Small-interfering RNA (siRNA)

Three predesigned combinations of 4 siRNAs duplex directed against rat DHCR24 mRNA (NM_053272) and Silencer Negative Control 1 (non-targeting siRNA) were purchased from Dharmacon (Lafayette, United States). The target sequences of siDHCR24s are listed below:

5'-GCACAGGCATCGAGTCATC-3'; 5'-GCACGGGTTCCAAATGTTA-3'; 5'-GCGCCTGGGTGGTGTTCAA-3'; 5'-ACTCAGACCTGTTCTATGC-3'.

The PC12 cells were seeded into 6-well plates 24 h before transfection. The siRNA (final concentration 50 nM) was introduced into the cells by the DharmaFECT1 siRNA transfection Reagent (Dharmacon; Lafayette, United States).

### Statistical analysis

Statistical analyzes was performed by one-way analysis of variance (ANOVA), followed by Bonferroni's multiple *t*-tests, with *p* < 0.05 considered to be statistically significant.

## Results

### DHCR24 displays low expression in AD brains

**Fig 1:**
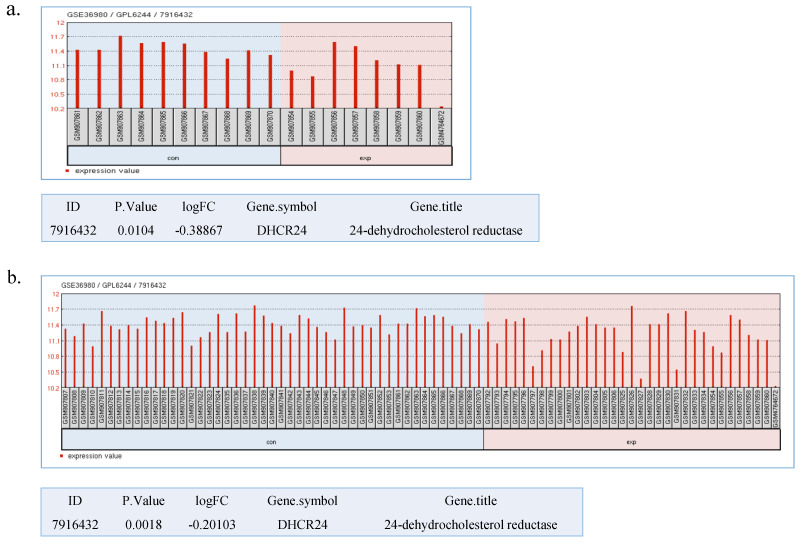
** Different expression of DHCR24 extracted from GSE36980.** (a) The expression of DHCR24 between AD patients and controls in the hippocampi. (b) The expression of DHCR24 between AD patients and controls in the hippocampi, frontal as well as temporal cortices. con: Non-AD; exp: AD. Statistical analysis in GEO2R was performed by using the Bayes test. *p* < 0.05

*DHCR24* has long been considered as an AD-related gene. Greeve *et al*. reported that the DHCR24 expression is decreased in the impaired region of the AD patients' brain 6. Recently, a study conducted microarray analyzes with RNAs obtained from the post-mortem AD brains 15. Here, we performed a biostatistical analysis on the mRNA expression level of DHCR24 taken from a publicly available database. The DHCR24 expression pattern is depicted in Figure 1. The mRNA level of DHCR24 was significantly decreased in the hippocampus region of 7 AD patients' brains when compared with that of 10 control samples (Fig. 1a). A similar reduction in the level of DHCR24 was evident in the frontal cortex and temporal cortex in 34 AD patients (Fig. 1b). These data again emphasize the relevance and importance of DHCR24 in the pathological mechanism of AD. Subsequent experiments were performed to verify the possible role of suppressing DHCR24 in the cholesterol biosynthesis and the IGF-1-Akt survival signaling pathway.

### Anti-apoptotic action of IGF-1 in PC12 is blocked via the DHCR24 inhibitor U18666A

**Fig 2:**
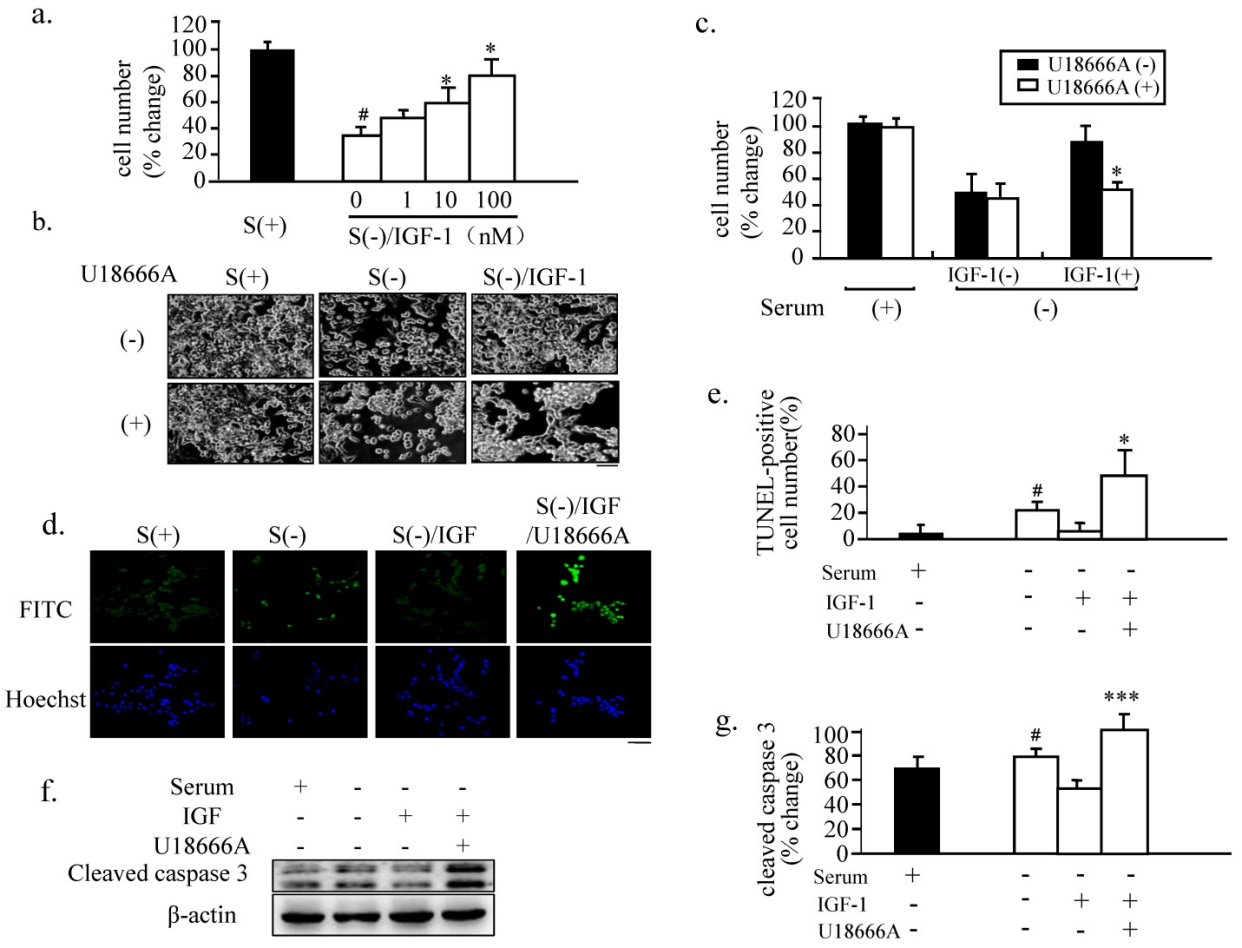
** Blocking of the neuron-protective function of IGF-1 in PC12 cells via U18666A treatment.** (a). The PC12 cells were cultured in complete growth medium [S(+)] or in a serum-free medium [S(-)] with the indicated dose of IGF-1 for 48 h. The numbers of adherent cells from each group were then counted. Data indicates the means ± SDs from 3 different experiments. '#' indicates significantly different from S(+) at *p* < 0.05; and '*' indicates significantly different from S(-)/IGF-1 at *p* < 0.05. (b). The PC12 cells were cultured in S(-)/IGF-1 without or with U18666A for 48 h. The images of the cells were obtained with a phase-contrast microscope 48 h after the U18666A treatment at the concentration of 1 μg/mL. This U18666A concentration was used in the subsequent cellular experiments. Scale bar = 50 μm. (c). The number of adherent cells from panel (b) was counted. Data indicates the means ± SDs from 3 separate experiments. '*' indicates significantly different from U18666A (-)/IGF-1(+)/S(-) at *p* < 0.05. (d). The PC12 cells were treated as in panel (b) for 48 h. DNA fragmentation was analyzed by the TUNEL assay and the nuclei were stained with Hoechst. Scale bar = 50 μm. (e). The percent of apoptotic cell numbers to the adherent cells indicated in panel (d) was determined 48 h after the U18666A treatment. Data indicate the means ± SDs from 3 separate experiments. '#' indicates significantly different from S(+) at *p* < 0.05 and '*' indicates significantly different from S(-)/IGF-1(+) at *p* < 0.05. (f). The PC12 cells were treated as in panel (b) for 48 h. Whole-cell lysates (30 μg/lane) were subjected to western blotting using anti-cleaved caspase 3 and anti-actin antibodies. The representative results are displayed. (g). A plot displaying the intensities of the bands in the blot shown in panel (f). The levels of cleaved caspase-3 were normalized against the level of actin and expressed as percent of the highest level of the groups. Data indicates the means ± SDs of 4 experiments. '^#^' indicates significantly different from the result of S(+) at *p* < 0.05. '***' indicates significantly different from the result of S(-) containing IGF-1 without U18666A at p < 0.001.

Given that FBS can provide an exogenous supply of cholesterol to the cells when cultured in the serum-containing growth medium and that cholesterol *de novo* synthesis can occur in the cells cultured with serum-free medium, we first subjected PC12 cells to serum deprivation to decrease the exogenous cholesterol supply and avoid the stimulation of growth hormones in FBS. We found that under this condition, the numbers of adherent cells were significantly reduced compared to the control cells cultured in FBS-containing medium (Fig. 2a). The PC12 cells treated with increasing concentrations of IGF-1 in the serum-free medium exhibited a steady increase in the number of adherent cells, with 10 nM of IGF-1 achieving approximately 60% adherence of the respective control value. Thus, 10 nM of IGF-1 was used in the subsequent experiments.

U18666A, 3-β-[2-(diethylamine) ethoxy] androst-5-en-17-one, is an amphipathic steroid. It is widely used to block the intracellular trafficking of cholesterol and to mimic the Niemann-Pick type-C disease 15, a hereditary lysosomal storage disease. U18666A blocks the traffic of free cholesterol from the late endosomal compartment. The administration of U18666A at a low dosage causes the accumulation of cholesterol in the late endosomes and lysosomes 16. U18666A also inhibits the cholesterol biosynthesis by inhibiting oxidosqualene cyclase and desmosterol reductase at a high dose 17, 18. In our preliminary experiment, we tested the U18666A concentrations in serum-free medium of cultured cells to evaluate its cytotoxicity (data not shown) and to decide whether the relative high dose of 1 μg/mL of U18666A can be used to inhibit the biosynthesis of cholesterol in subsequent cellular experiments.

When the IGF-1-treated cells were cultured in a serum-free medium and exposed to a relative high dose of U18666A, the neuron-protective action of IGF-1 was found to be significantly attenuated (Fig. 2b), resulting in the detachment of approximately 50% of the cells on exposure to U18666A for 18 h than when the cells were treated with IGF-1 only (Fig. 2 c). U18666A alone did not markedly affect the number of attached cells when compared with the serum-withdrawal cells, indicating the minimal cytotoxicity produced by 1 μg/mL of U18666A. Treatment with IGF-1 markedly increased the number of attached cells, and U18666A reversed this effect of IGF-1. TUNEL staining suggested that cell death induced by serum deprivation was mainly due to apoptosis (Figs.2d and e). Western Blot analysis also showed that the activation of caspase-3 induced by serum deprivation was prevented by IGF-1 treatment, and U18666A blocked this effect of IGF-1. These results demonstrated that treatment of the serum-deprived cells with IGF-1 prevented serum deprivation-induced apoptosis, while U18666A blocked the neuron-protective function of IGF-1.

### U18666A inhibits the DHCR24 activity in cholesterol biosynthesis and impairs the caveolae structures

**Fig 3:**
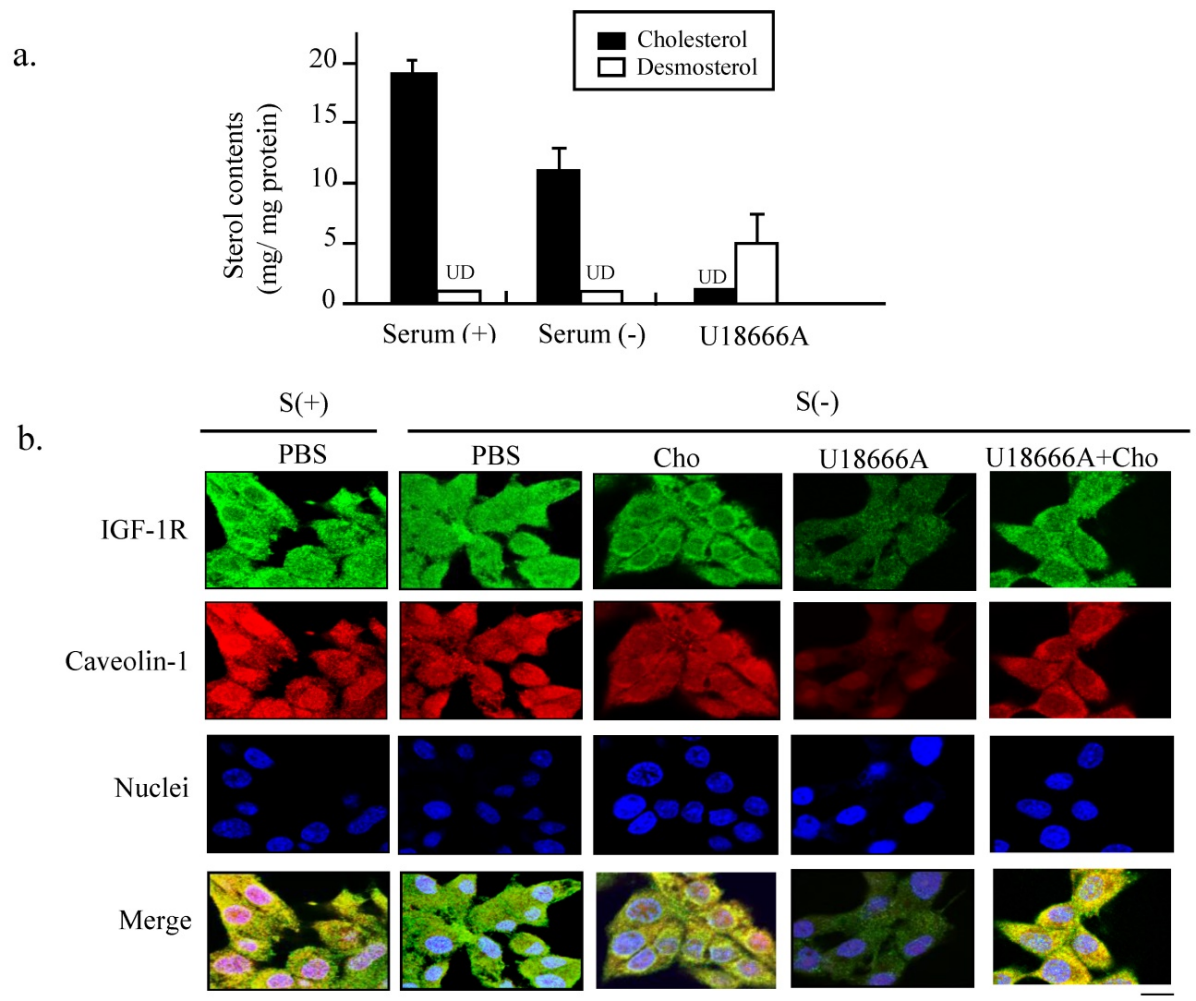
** Depletion of cellular cholesterol and impaired localization of IGF-1 receptor in caveolae induced by U18666A.** (a). The PC12 cells were cultured in the absence and presence of serum for 24 h. The medium was extracted and subjected to HPLC analysis. Cellular cholesterol and desmosterol contents were indicated by filled and unfilled columns, respectively. Data indicate the means ± SDs from 3 separate experiments. UD, Undetectable. (b). Immunocytochemical analysis was performed using the PC12 cells cultured in a growth medium (S+) or in a serum-free medium with or without U18666A. In some groups, the cholesterol (40 μg/ml) added into the cells cultured in the serum-free medium with or without U18666A. The cells were probed with rabbit anti-caveolin-1 antibody and mouse anti-IGF-1R antibody, followed by a mixture of anti-mouse IgG antibody conjugated with Alexa fluor-488 and anti-rabbit IgG antibody conjugated with Alexa fluor-568. Images were obtained using a confocal laser microscope. The red and green fluorescent signals represent caveolin-1 and IGF-1R proteins, respectively. Scale bar = 5 μm.

To confirm the inhibitory effect of U18666A on the cholesterol biosynthesis, we performed HPLC analysis to determine the contents of intracellular cholesterol and desmosterol of PC12 cells after U18666A exposure. As shown in Figure 3a, the levels of cholesterol and desmosterol were not significantly different from each other when the cells were subjected to serum deprivation. However, exposure to U18666A significantly decreased the cholesterol level, resulting in the accumulation of desmosterol. This data suggested that treatment of PC12 cells with U18666A could inhibit the DHCR24 activity, thereby preventing the enzyme from converting desmosterol to cholesterol.

To further explore why the U18666A-induced cholesterol loss blocked the neuron-protective functions of IGF-1, we performed immunocytochemical double staining to observe the structure of cholesterol-rich microdomain on the plasma membrane, caveolae. As discovered in our previous study the caveolae are present on the plasma membrane of PC12, which are required for maintaining the IGF-1-Akt cell survival signaling 10. The green fluorescent signals representing the IGF-1R and red signal representing caveolin-1 in Figure 3b showed similar locations after merging in the cells cultured in a complete medium, suggesting that IGF-1 receptor was localized in the caveolae, which was consistent with our previous finding. This co-localization of IGF-1 receptor with caveolae was weakly diffused in the cells cultured in serum-free medium, while cholesterol addition into the medium prevented the destruction of the co-localization. However, the intensity in both luminance and the brightness of both green and red fluorescent signals became significantly weaker in the S(-)/U18666A group. The merged yellow signals were significantly more diffused in the U18666A-treated cells, suggesting that U18666A-induced cholesterol loss impaired the formation of the caveolae structure and the localization of IGF-1 receptor in caveolae via depletion of the cellular cholesterol storage. The supplement of cholesterol into the cells cultured in the serum-free medium with U18666A significantly prevented the effect of U18666A on the disruption of caveolae structure and impairment of co-localization of IGF-1 receptor with caveolae. These data suggested that the cholesterol loss induced by U18666A could impair the structure of the cholesterol-rich microdomain caveolae on the plasma membrane and disrupt the co-localization of IGF-1 receptors with caveolae in PC12 cells.

### siRNA against DHCR24 blocks the neuron-protective function of IGF-1

**Fig 4:**
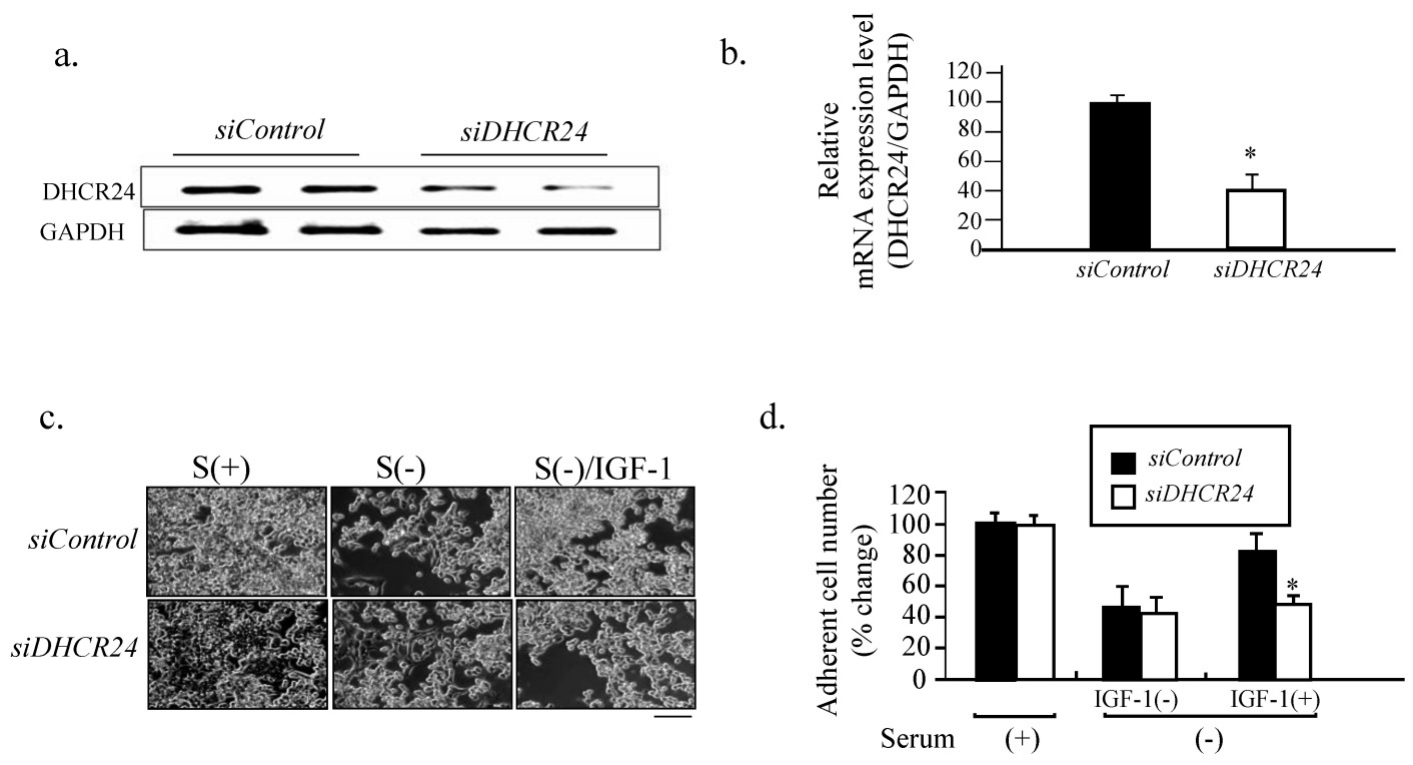
** Blocking of the neuron-protective function of IFG-1 by DHCR24 RNA interference.** (a). The PC 12 cells were cultured in a growth medium and transfected with siDHCR24 or siControl for 48 h and then subjected to semi-quantitative PCR with the DHCR24-specific primers and GAPDH-specific primers, followed by an agarose gel electrophoresis analysis. (b). The relative mRNA expression (DHCR24/GAPDH) obtained from panel (a) were expressed as means ± SDs from 3 separate experiments. '*' indicates significantly different from siControl at *p* < 0.05. Similar results were obtained from 3 separate experiments. (c). The PC12 cells were transfected with siDHCR24 and siControl for 48 h, followed by serum starvation without or with IGF-1. Cell images were obtained with a phase-contrast microscope at 48 h after U18666A treatment. Scale bar = 50 μm. (d). The number of adherent cells from panel (c) was counted. The adherent cell number in the cells cultured in the medium containing FBS [siControl in Serum(+)] was counted as 100%. Data indicate the means ± SDs from 3 experiments. '*' indicates significantly different from siControl/Serum (-)/IGF-1(+) at *p* < 0.05. Similar results were obtained from 3 separate experiments.

To avoid possible off target effects of U18666A in addition to its inhibition of the DHCR24 activity, we downregulated the level of DHCR24 l in PC12 cells using siRNA, followed by investigation of the neuron-protective function of IGF-1 against serum withdrawal-induced cell detachment. Semi-qualitative PCR of the DHCR24 mRNA level revealed that it was significantly decreased in PC12 cells transfected with siDHCR24 when compared with cells transfected with siControl (Figs. 4a and b). The number of attached cells decreased in response to serum withdrawal for both PC12 cells transfected with siDHCR24 and those transfected with siControl. However, there was a further reduction in the number of attached siDHCR24-transfected cells when supplemented with IGF-1 in comparison to cells transfected with siControl, (Figs. 4c and d). These results suggested that targeting DHCR24 with siRNA could also block the neuron-protective function of IGF-1 in PC12 cells.

### Loss of cholesterol induced by U18666A impairs the IGF-1-Akt-Bad signaling in PC12 cells

**Fig 5:**
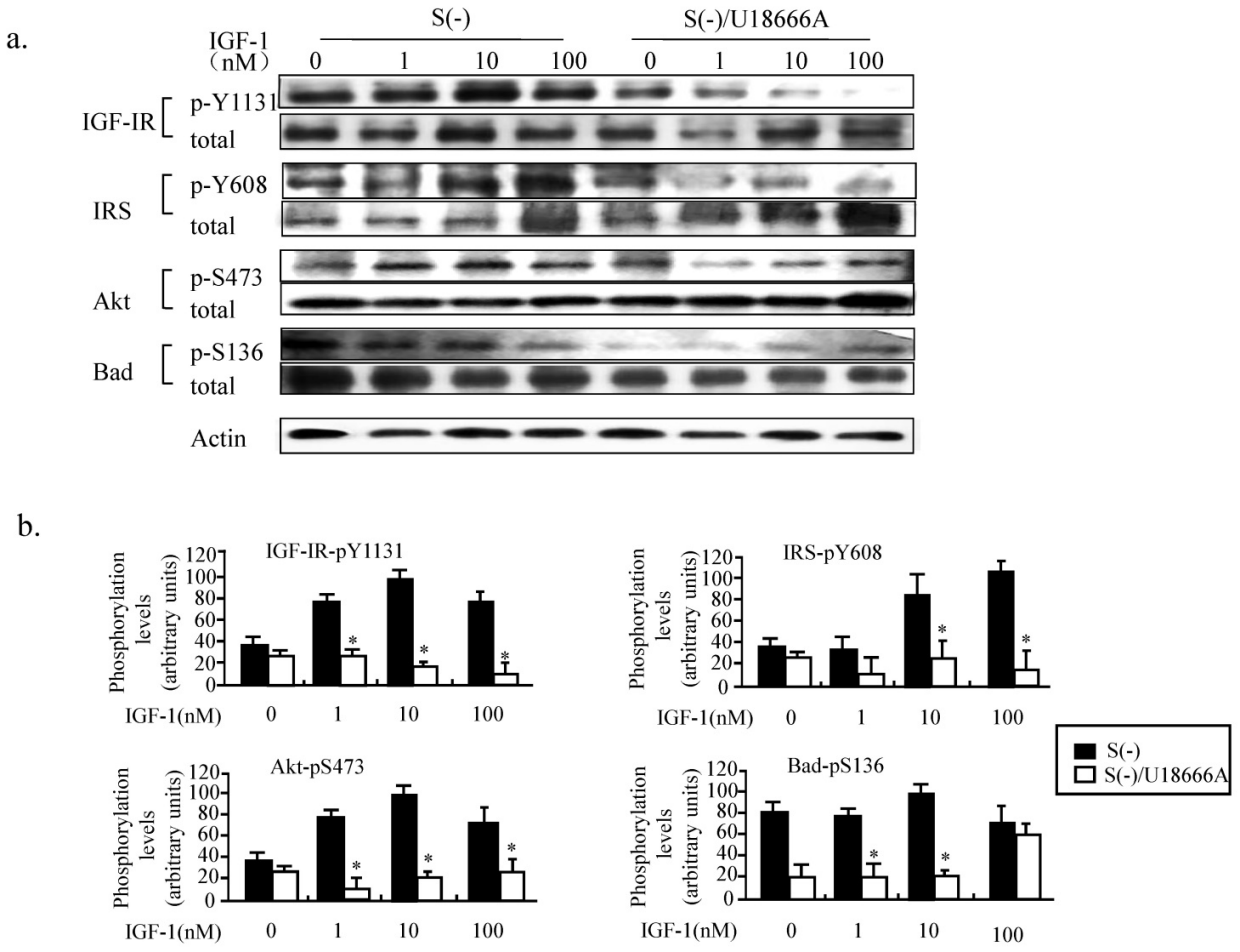
** Impairment of IGF-1 functions in PC12 cells caused by U18666A-induced depletion of cellular cholesterol.** (a). Western blotting showing changes in the phosphorylation levels of IGF-1R, IRS, Akt, and Bad in the PC12 cells cultured in serum-free medium with or without U18666A for 14 h following treatment with 10 nM IGF-1 for 30 min. Whole-cell lysates (30 μg/lane) were subjected to western blotting using anti-phospho IGF-1R (p-Y1131), anti-IGF-1R, anti-phospho-IRS (p-Y608), anti-IRS, anti-phospho-Akt (p-S473), anti-Akt, anti-phospho-Bad (p-S136), anti-Bad and anti-actin antibodies. The representative results are displayed. (b). A plot displaying the intensities of the bands in the blot shown in panel (a). The levels of phosphorylated proteins were normalized against the level of each total proteins and expressed as percent of the highest level of the phosphorylated form for of each group. Data indicates the means ± SDs of 4 experiments. '*' indicates significantly different from the result of S(-) without U18666A at *p* < 0.05.

Both insulin and IGF-1 bind to insulin receptors and IGF-1 receptors and activate the PI3K/Akt signaling pathways in the brain 19. IGF1 crosses the blood-brain barrier with significantly greater efficiency than insulin, and the IGF-1-Akt signaling has been shown to be necessary and sufficient for neuron survival 19. We therefore studied the phosphorylation status of the key proteins involved in the PI3K/Akt signaling pathway, including the IGF-1 receptor, IRS, Akt, and Bad. As shown in Figure 5, when the PC12 cells cultured in serum-free medium without and with U18666A were treated with IGF-1 for 30 min, the IGF-1-Akt-Bad signaling pathway gets activated, and, in an IGF-1 dose-dependent manner as revealed by the increased phosphorylation of the various key proteins of the PI3K/Akt signaling pathway. However, treatment with U18666A in serum-free medium could significantly inhibit the phosphorylation of IGF-1 receptor, IRS, Akt, and Bad in response to IGF-1 stimulation within 30 min, while the total level of these proteins seems to be somehow upregulated after IGF-1 stimulation. This result suggested that cholesterol depletion induced by U18666A impaired the IGF-1-Akt-Bad cell survival signaling.

### Intracerebral U18666A decreases the cholesterol level and impairs the IGF-1-Akt signaling in the brain

To further understand the role of cholesterol in the IGF-1-Akt signaling *in vivo*, relevant animal experiment was performed. The rats were administered with U18666A via intracerebral injection. Both size and weight of the brains taken from the control (PBS) and U18666A-treated groups were not significantly different (data not shown). We thus examined how the underlying molecular mechanism of cholesterol loss can impair the IGF-1-Akt signaling pathway *in vivo*.

**Fig 6:**
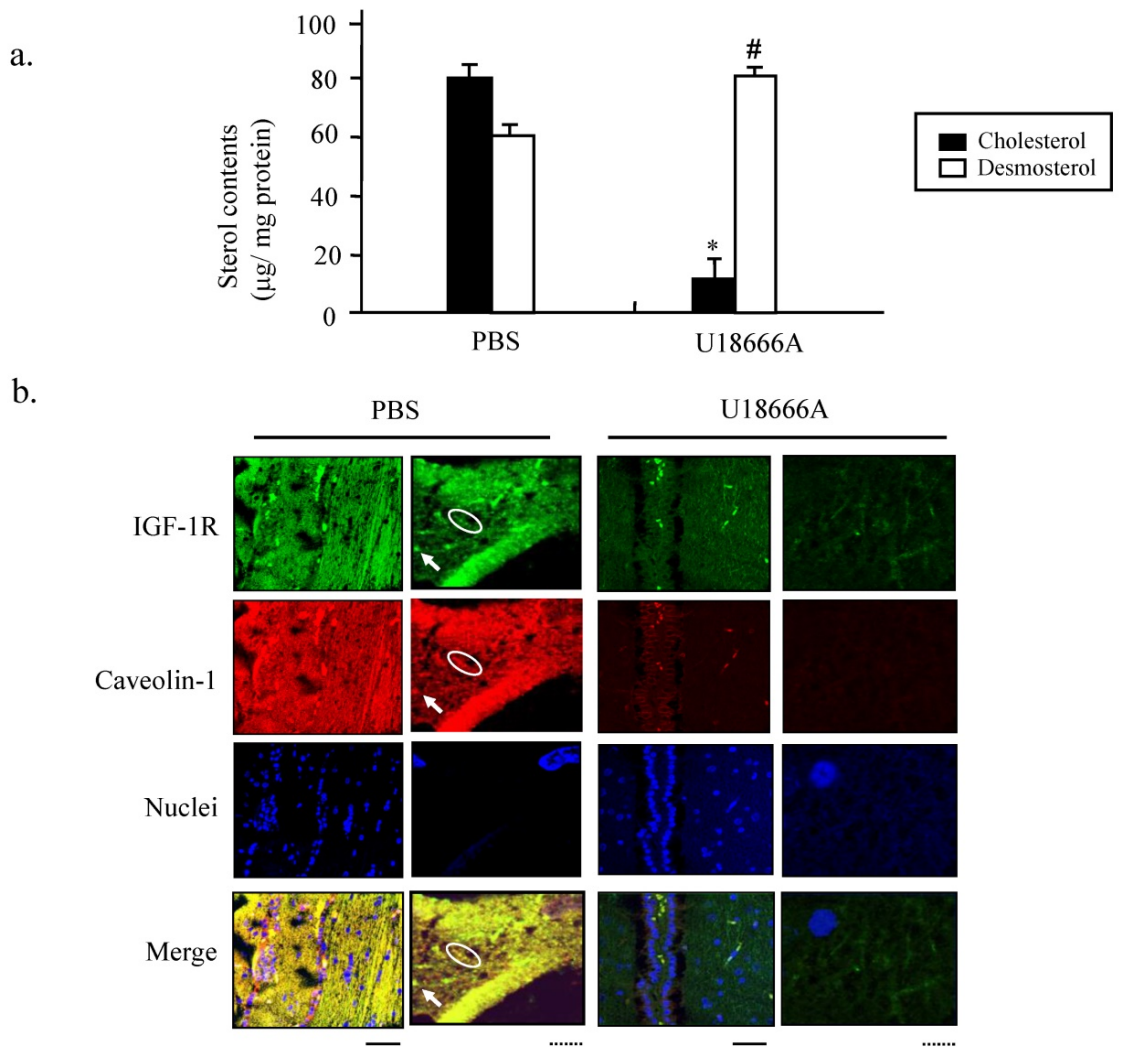
** Depletion of cholesterol and accumulation of desmosterol in the temporal lobes of rat brain induced by ICV injection of U18666A.** The SD rats were administered by ICV injection of PBS or U18666A and then sacrificed after 0 (PBS control group) or 14 days (U18666A-injected group). The temporal lobes portion were separated and subjected to the HPLC assay or immunohistofluorescent analyzes. (a). The cellular cholesterol and desmosterol contents were measured by HPLC and represented by closed and open columns, respectively. Data indicates the means ± SDs from 8 rats. '*' and '#' indicates, respectively, the cholesterol and desmosterol levels were significantly different from those of the control at *p* < 0.05. (b). The localization of IGF-1 receptor in the caveolae of the hippocampus as demonstrated by immunohistochemistry. Immunohistochemistry was performed using antibodies against the caveolin-1 and IGF-1 receptors. The slides of hippocampus obtained from rats were fixed with paraffin and then probed with rabbit anti-caveolin-1 antibody and mouse anti-IGF-1R antibody, followed by a mixture of anti-mouse IgG antibody conjugated with Alexa fluor-488 and anti-rabbit IgG antibody conjugated with Alexa fluor-568. The images were obtained using a confocal laser microscope. The red and green fluorescent signals represent caveolin-1 and IGF-1R proteins, respectively. Scale bar represented by solid line is 100μm and dotted line is 10 μm.

First, the contents of cholesterol and desmosterol in the brain tissues of both the groups of rats were determined. As shown in Figure 6a, the cholesterol level was strongly decreased, while the desmosterol content was significantly increased in the ic-U18666A group when compared with that in the control group. This result demonstrated that ic-U18666A induced the depletion of cholesterol and accumulation of desmosterol by blocking the DHCR24 activity. Double immunocytochemical analysis with the antibodies against IGF-1R and caveolin1 was conducted to examine the localization of IGFR in the caveolae in the brain tissues of the temporal lobe (Figure 6b). Adequate merging of the green fluorescent signal representing IGF-1R and the red fluorescent signal representing caveolin1 were obtained from PBS group. Inspection of the magnified picture enabled visualization of the merging of the green spots with the red spots, yielding yellow spots for almost every spot. This result thus demonstrated, for the first time, that the IGF-1 receptor is located in the caveolae of rat brain. However, both of green and red fluorescent signals from the brain slices of rats with U18666A treatment for 14 days became very diffused and darker, and it was very difficult to find the yellow spots representing the well co-localization of IGF-1R with caveolae. These data suggested that U18666A treatment could decrease the cellular level of cholesterol and impair the structure of caveolae and co-localization of IGF-1R with caveolae in the brain of rats.

**Fig 7:**
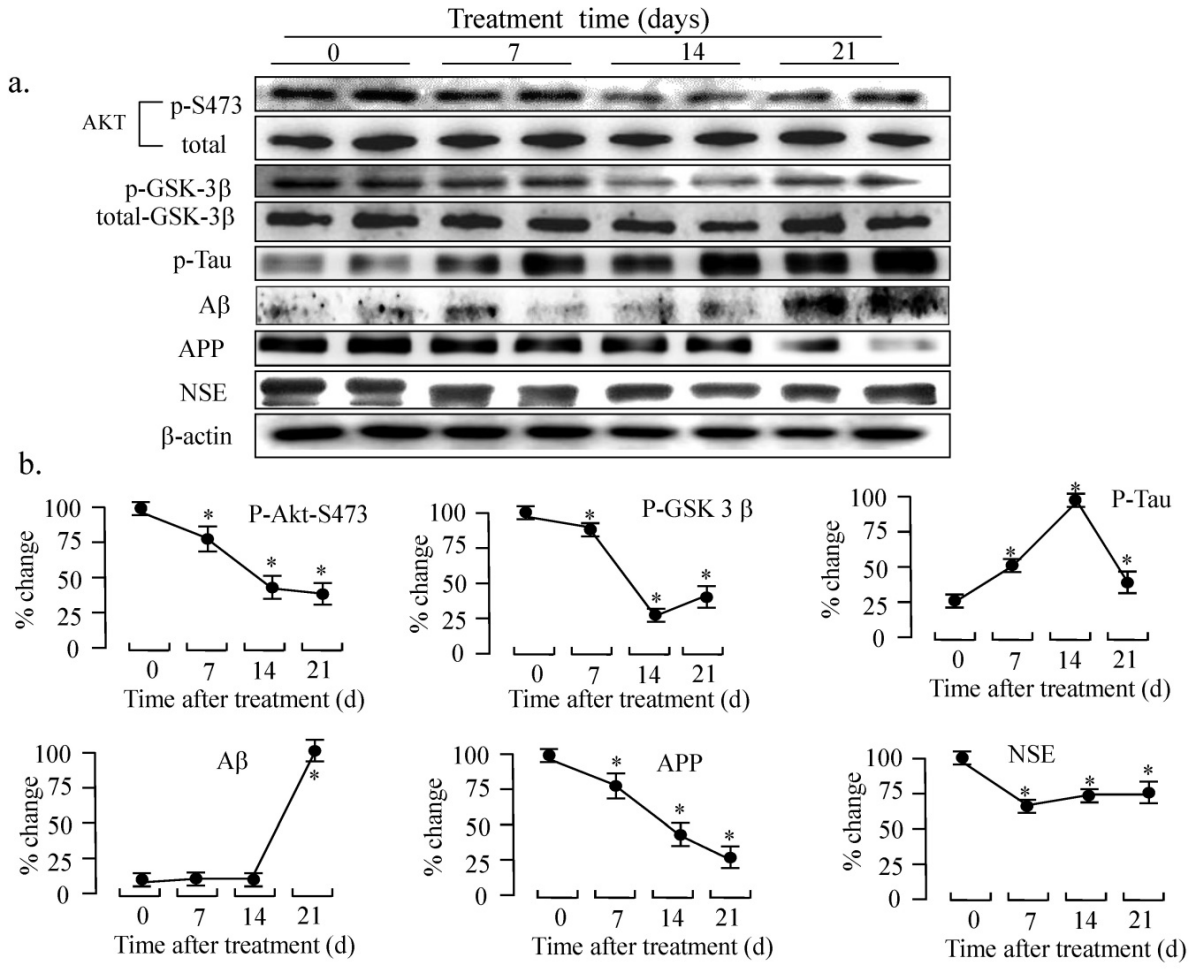
** Intracerebroventricularly injected U18666A impairs the IGF-1-Akt signaling and induces an accumulation of amyloid β in the temporal lobes.** (a). Western blotting demonstrating changes in the levels of expression or phosphorylated status of the indicated proteins. Rats were intracerebroventricularly injected with U18666A or PBS at 0, 7, 14, or 21 days and then sacrificed. The total proteins were extracted from the tissues of the temporal lobes (30 μg/lane) and then subjected to western blotting with anti-phospho-Akt (p-Ser473), anti-Akt, anti-phospho-GSK-3β (p-Ser-9), anti-GSK-3β, anti-phospho-Tau (p-Ser404), anti-Tau, anti-Aβ, anti-NSE, and anti-actin antibodies. (b). A plot showing the intensities of the bands in the blot depicted in panel a. The levels of phosphorylated proteins were normalized against the level of each total proteins, and then expressed as a percent of the highest level of the phosphorylated form for each group. Data are indicated as the mean ± SDs from 8 rats. '*' indicates significantly different from the level at day 0 at *p* < 0.05.

GSK3β is one of the key molecules downstream of the IGF-1 signaling pathway that has been shown to be involved in the formation of pathomorphological AD hallmarks, such as Aβ plaques, hyperphosphorylated Tau, and cerebral neuronal death^20^, ^21^. Next, we analyzed the IGF-1-Akt signaling and the accumulation of Aβ in the animal model by western blotting. As shown in Figure 7, the phosphorylation of Akt and GSK-3β was significantly decreased after ic-U18666A treatment for 14 days, suggesting that the activity of Akt was impaired and the activity of GSK3β was improved by U18666A treatment. Increased levels of phospho-Tau have been associated with AD, while Tau hyperphosphorylation has been linked to increased GSK-3β activity. Furthermore, hyperphosphorylation of Tau protein in the hippocampus of central insulin-resistant rats has been linked to cognitive impairment 22. Here, we examined the effect of U18666A treatment on the phosphorylation level of Tau. The phosphorylation of Tau reached a peak after treatment for 14 days. These changes in the phosphorylation of Akt, GSK-3β, and Tau remained significant until day 21 of the treatment. Interestingly, although the phosphorylation of these proteins was subsequently reduced, we could still observe significant accumulation of Aβ and the decrease in APP in the U18666A-treated rats.

The neuron-specific enolase (NSE) has been widely applied and accepted as a neuron molecular marker in the studies of neurodegeneration disease 23. As shown in Figure 7, the expression of NSE also decreased as early as 7 days after the treatment with U18666A and it continued to decrease until 21 days of the treatment. This result suggests that the number of neurons in the temporal lobe of the rat brain was decreased in response to the U18666A treatment.

**Fig 8:**
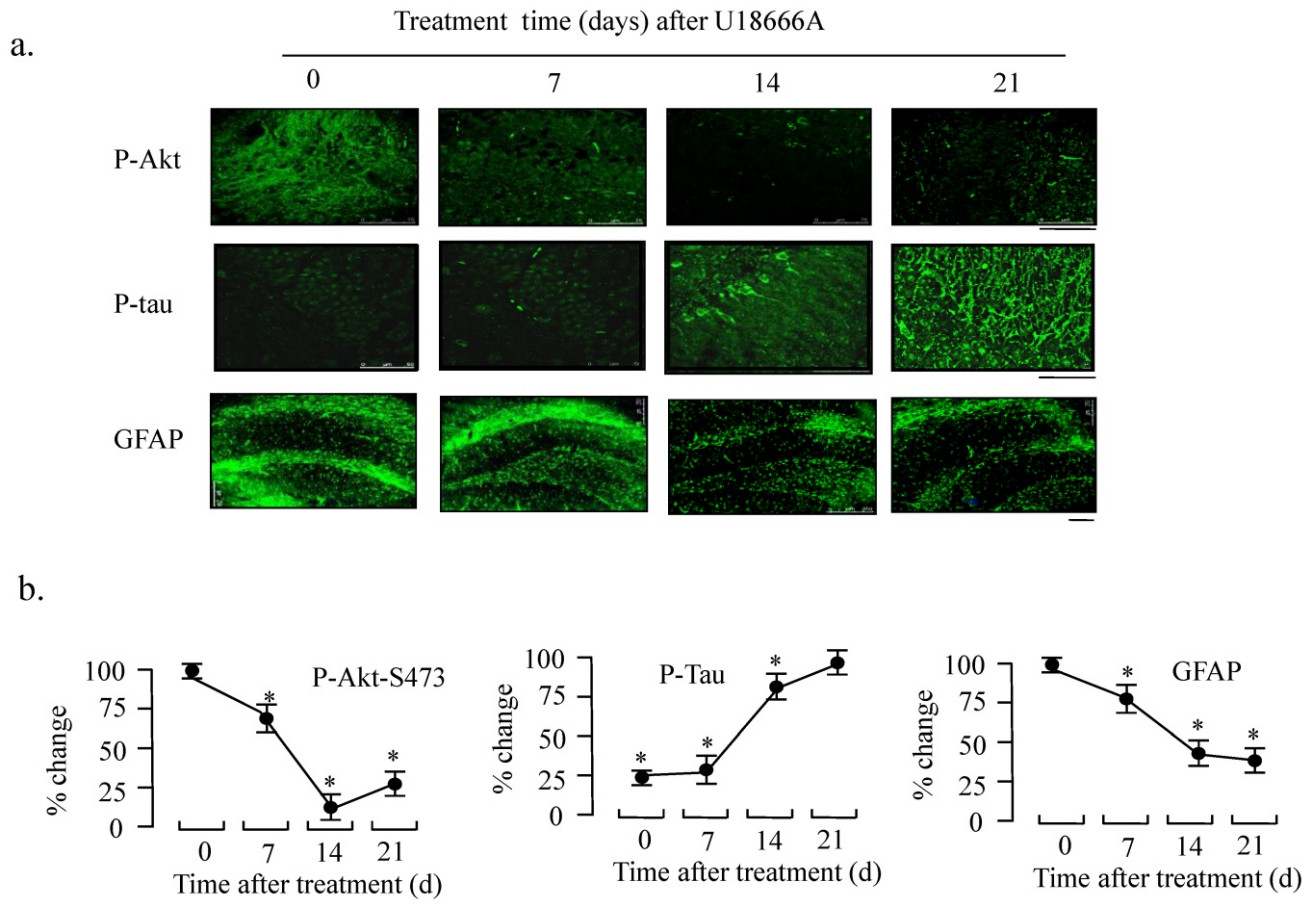
** Effect of ICV injection of U18666A on the phosphorylation levels of Akt and Tau in rat brains.** Rats were intracerebroventricularly injected with U18666A for 0, 7, 14, or 21 days and then sacrificed. The slides were prepared from the temporal lobes and fixed for immunohistofluorescent analysis with anti-phospho-Akt (p-Ser473), phospho-Tau (p-Ser9), and anti-GFAP as first antibodies and the second IgG antibodies conjugated with Alexa 568. The images were obtained using a confocal laser microscope, followed by densitometric analysis. Data are indicated as the means ± SDs from 8 rats. '*' indicates significantly different from the level at 0 time at *p* < 0.05. Scale bar = 75 μm (p-Akt), 50 μm (p-tau), and 250 μm (GFAP).

In support to the western blotting results, immunocytochemical analysis also demonstrated decreased phosphorylation of Akt and increased phosphorylation of Tau in response to U18666A treatment in the temporal lobe at days 7, 14, and 21 (Fig. 8). Furthermore, we studied the effect of U18666A treatment on the GFAP expression because it is one of the most widely used markers of astrocyte which is the most widely distributed one of the glial cells. In addition, in Figure 8 (lowest panel), the expression of GFAP can be seen to be significantly decreased after treatment with U18666A for 14 and 21 days, suggesting that cholesterol depletion induced by U18666A may have impaired the survival of glial cells.

### U18666A treatment may impair the spatial memory of rats in Morris water maze

**Fig 9:**
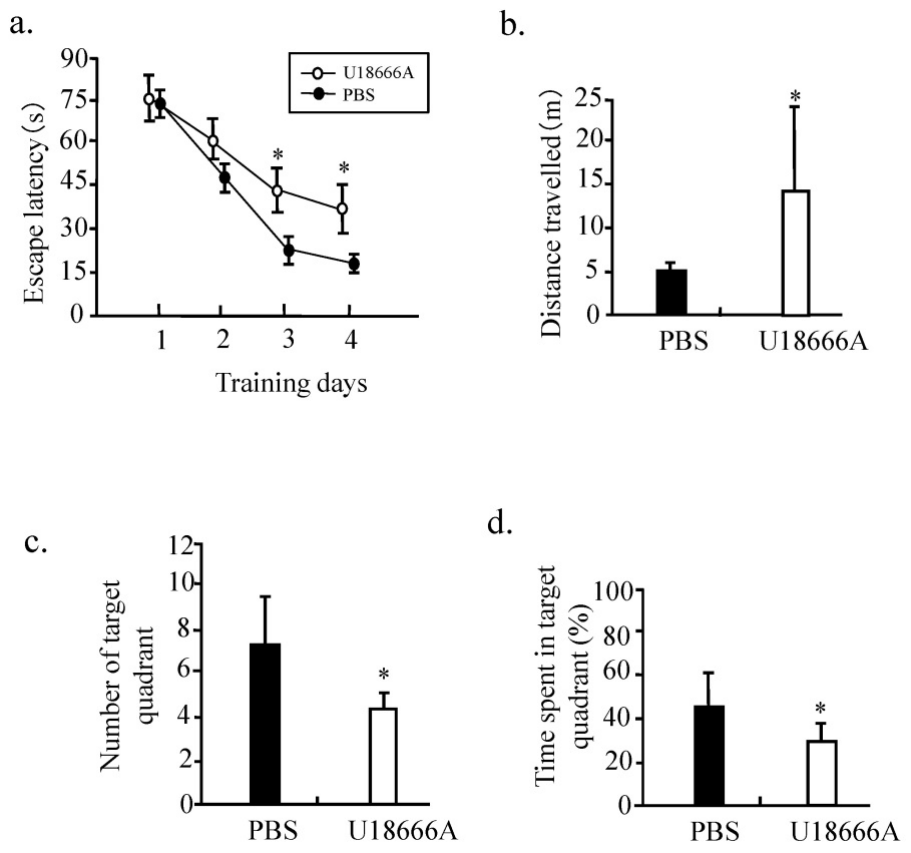
** Spatial learning impairment in SD rats treated with icv-injection of U18666A.** Morris water maze tests included 4 days of visible platform training and 1 day of hidden platform testing after 21 days of receiving the ICV injection of U18666A or PBS. (a) Bar graphs representing the escape latency acquired by rats on the first 4 platform training days. (b) Bar graphs representing the distance travelled acquired by the rats on the fifth testing day. (c) and (d) Bar graphs representing the number of target quadrants crossing and time spent in target quadrant in the spatial probe test on the fifth testing day. Data indicate the means ±SDs from 8 rats. '*' indicates significantly different value from the control value at *p* < 0.05.

The Morris water maze experiment was conducted to investigate whether U18666A treatment could result in the cognitive deficits in the rats. The results obtained from the learning curve shown in Figure 9 indicated that that the mean escape latency for the trained rat model of the U18666A group to search for the visible platform was increased in comparison to that of the control. U18666A-treated rats also travelled significantly longer distance to find the platform relative to the control rats (Fig. 9b). This result demonstrated that the spatial learning skills of rats were impaired by the U18666A treatment. Similarly, the significant differences in the number of target quadrant and time spent in the target between the ic-U18666A and control groups were also observed (Fig. 9c and d). Cumulatively, these results suggest that U18666A treatment can cause cognitive deficit in rats.

## Discussion

The results of the present study demonstrated that cholesterol depletion induced by blocking the activity of DHCR24 (Fig. 2) or by downregulating the expression of DHCR24 (via RNA interference technology) in neuronal cells reduced the adherent cell numbers (Fig. 4). The blocking of the DHCR24 activity by its inhibitor U18666A resulted in decreased cellular cholesterol level and hampered the functions of caveolae for the recruitment of IGF-1 receptor (Fig. 3). The activation of IGF-1-Akt signaling in response to IGF-1 was also impaired in U18666A-treated cells (Fig. 5). The lack of DHCR24 expression has previously been demonstrated to increase the susceptibility of mouse embryonic fibroblast (MEF) to serum deprivation-induced apoptosis through decreasing cellular cholesterol content, thereby impairing the insulin-Akt-Bad signaling. The present data further confirmed the role of DHCR24 in maintaining the caveolae structure and the insulin/IGF-1 signaling pathway in neuronal cells.

In the recent years, several studies have proposed that AD could be a “Type 3 diabetes” disease 24-29. Increasing evidence supports this theory. In the neuronal tissues, the insulin receptor system activates several enzymes of glucose metabolism, suggesting that, in the CNS, insulin may also play an important role in the glucose metabolism 30. Both insulin and IGF-1 promote neurite outgrowth, synapse formation, and neuronal survival 31. Both insulin and IGF-1 activate the PI3k/Akt pathways in the brain 19. Moreover, accumulated evidence has demonstrated that deficient glucose utilization and energy metabolism occur early, which in turn suggest a role of the impairment of insulin signaling in AD^26^, ^32^-^34^. Extensive abnormalities in the signaling mechanisms of insulin and insulin-like growth factor types I and II (IGF-I and IGF-II) have been detected in the brains of AD patients 25, 26, 32-34. For example, the expression levels of IGF receptors are significantly decreased in AD. The significantly decreased expression of genes encoding insulin, IGF-I, and IGF-II, as well as the insulin and IGF-I receptors in the CNS suggested that AD may be a type 3 diabetes in the brain, yet it is different from diabetes mellitus 26. This theory was proved once again by a study on an animal model in which intracerebral streptozotocin was used to deplete the brain insulin content 35. Recently, a new study revealed the function of a triple receptor agonist (TA) in activating glucagon-like peptide-1 (GLP-1), glucose-dependent insulinotropic polypeptide (GIP), and glucagon receptors at the same time in animal models of AD. This study also showed that TA treatment could significantly improve the memory impairment of APP/PS1 mice 36. Past clinical studies have also shown that intranasal insulin therapy may exert a therapeutic effect on AD patients and on patients with mild cognitive impairment 37. These studies indicated that insulin-related cell survival signaling pathway is either impaired or abnormal in the brain of AD patients. However, it remains unknown how the insulin/IGF-1-related signaling has become abnormal.

We previously demonstrated that the maintenance of insulin/IGF-1 signaling requires a normal cholesterol level and a caveolae structure in neuronal cells. A recently published report demonstrated, for the first time, the existence of a relationship between the cholesterol level and Aβ processing in living humans 38, which is consistent with the earlier autopsy reports, epidemiological findings, and in vivo animal model and in vitro cell culture, together suggesting an important role for cholesterol in AD. In addition, early reports have also shown that the level of DHCR24 decreases in the affected brain areas of AD patients 6. The downregulation of DHCR24 may result in the disorganization of detergent-resistant membrane domains (DRM) and in the increased formation of Aβ42 though decreasing the biosynthesis of cholesterol in the brain 39. Published clinical data indicates that several single nucleotide polymorphism sites (SNPs) in DHCR24 possess significantly different gene frequencies between the AD patients and control 40. These SNPs are located in the 3'-UTR or intron regions of *DHCR24*, suggesting that the abnormal expression of DHCR24 may contribute to the pathological mechanism of AD. The expression of DHCR24 in the brain gradually decreases with aging. The accumulation of the cholesterol precursor desmosterol and the reduced expression of DHCR24 have also been reported in 21-month-old APPSLxPS1mut mice accompanied by AD-specific abundant amyloid deposits 41. These studies together suggest a possible relationship among DHCR24, cellular cholesterol level and insulin/IGF-1 signaling in the brain. Our data provides new insight into this mechanism, one by which decrease in the DHCR24 expression in the affected brain areas of AD patients may induce cholesterol loss and thus impairment in the insulin/IGF-1 cell survival signaling, which could eventually lead to the onset of apoptosis for several neurons.

To further demonstrate this possibility, we studied the effect of U18666A on the function of rat brain. We found that the U18666A treatment also impaired the activation of IGF-1-Akt signaling, including increased activation of Tau and the accumulation of Aβ (Fig. 7). The spatial memory of U18666A-treated rats was also partially impaired in comparison with the control (Fig. 9). We first observed the presence of caveolae in the hippocampus area and the localization of IGF-1 receptor in the caveolae in U18666A-treated rats, which suggests that the IGF-1 signaling pathway may require the normal structure and function of caveolae (Fig. 6). This observation was consistent with those of our previous study demonstrating a similar mechanism in the central nervous system 10. Our data thus suggested that the U18666A treatment could induce the impairment in both neuron and glial cells, as demonstrated by the lower expressions of NSE and GFAP proteins (Figs. 7 and 8).

Several recently published papers have reported a lack of change in the DHCR24 expression in the brain of AD patients (Fig. 1). We believe that the pathological mechanism of AD is extremely complicated as several factors can contribute to the pathology of AD. However, one early study has revealed significantly elevated insulin level in the plasma, but much lower insulin level in the cerebrospinal fluid (CSF) of AD patients relative to the controls. This imbalance may be because insulin does not function effectively in the brains of these patients 41, 42. In rats, Intracerebroventricular injection of streptozotocin or depletion of neuronal IR can also seriously impair the ability of these animals to remember a compartment in which they had received an electric shock 24, 27, 43-45. Interestingly, a recent study observed that diabetic mice have suppressed cholesterol biosynthesis in the brain due to the reduced expression of SREBP 46. These mice also have reduced levels of SREBP cleavage-activating protein (SCAP), which causes further impairment of cholesterol synthesis 47. Our data thus suggest that decreased cholesterol biosynthesis induced by the decreased expression of DHCR24 can result in an impairment of the insulin/IGF-1 signaling pathway, thereby leading to the impairment of synaptogenesis, neuronal function, and/or neuron death, all of which may contribute to the further impairment of the neurological functions, including AD.

Notably, this study did not demonstrate serious impairment by U18666A treatment with respect to memory trials in the animal model. One reason for this event can be that the impairment of the insulin /IGF-1 pathway may not be the only factor associated with the pathological mechanism of AD. Other possibilities such as oxidative stress, endoplasmic reticulum stress, and Aβ accumulation may also contribute to the occurrence and progression of AD 29, 48-52. Furthermore, according to several previous studies about the DHCR24 function 53, 54, DHCR24 may also possess multiple functions, such as protecting neurons against oxidative and ER stresses, and hence prevents the neurons from undergoing apoptosis. On the other hand, the rats in the present study were sacrificed after Morris water maze experiments on day 21 after the injection procedure. This duration may be so long that it removes U18666A from the brain; this observation is supported by the data obtained from western blotting analysis showing that the strongest effects of U18666A were observed 14 days after the injection, but not after 21 days (Fig. 7). Whether the overexpression of DHCR24 can reverse the memory impairment caused by AD in animal models could become an important part of future studies in this field. Taken together, our present reports clearly indicate that the loss of function of DHCR24 can induce the depletion of cholesterol and impair the IGF-1-Akt cell survival signaling in the central neuronal system.

We believe that our findings would provide a new explanation for an observation of AD patients with decreased DHCR24 expression of affected areas of brains that may induce neuronal apoptosis via impaired IGF-1-Akt survival signaling. Our present results also provide new evidence supporting the notion that AD may be type 3 diabetes. However, type 3 diabetes theory of AD is mainly based on the evidence that the impairment of insulin signaling in AD patients' brain is caused by a decrease in the insulin and IGF-1 receptor expression like type-1 diabetes 25, 26. Our data thus suggest that the neuron apoptosis in the affected areas of the AD patients' brain may be caused by the impairment of the insulin/IGF-1 signaling induced by the loss of cholesterol content and the dysfunction of caveolae, while the expression level of proteins of the insulin/IGF-1 signaling pathway may not be decreased. Taken together, our data may provide a new insight into the emergence of a new theory of AD, at least for some types of AD patients, which states that AD may be Type 4 diabetes that represents the impairment of the insulin/IGF-1 activity in the brain, although it needed further validation. Our results also provide a novel mechanism for the effectiveness of insulin therapy in AD patients 27, 35, 45.
